# Population-scale whole genome sequencing identifies 271 highly polymorphic short tandem repeats from Japanese population

**DOI:** 10.1016/j.heliyon.2018.e00625

**Published:** 2018-05-22

**Authors:** Satoshi Hirata, Kaname Kojima, Kazuharu Misawa, Olivier Gervais, Yosuke Kawai, Masao Nagasaki

**Affiliations:** a Graduate School of Medicine, Tohoku University, 2-1, Seiryo-machi, Aoba-ku, Sendai 980-8573, Japan; b Tohoku Medical Megabank Organization, Tohoku University, 2-1, Seiryo-machi, Aoba-ku, Sendai 980-8573, Japan; c Graduate School of Information Sciences, Tohoku University, 6-3-09, Aramaki Aza-Aoba, Aoba-ku, Sendai 980-8579, Japan

**Keywords:** Genetics

## Abstract

Forensic DNA typing is widely used to identify missing persons and plays a central role in forensic profiling. DNA typing usually uses capillary electrophoresis fragment analysis of PCR amplification products to detect the length of short tandem repeat (STR) markers. Here, we analyzed whole genome data from 1,070 Japanese individuals generated using massively parallel short-read sequencing of 162 paired-end bases. We have analyzed 843,473 STR loci with two to six basepair repeat units and cataloged highly polymorphic STR loci in the Japanese population. To evaluate the performance of the cataloged STR loci, we compared 23 STR loci, widely used in forensic DNA typing, with capillary electrophoresis based STR genotyping results in the Japanese population. Seventeen loci had high correlations and high call rates. The other six loci had low call rates or low correlations due to either the limitations of short-read sequencing technology, the bioinformatics tool used, or the complexity of repeat patterns. With these analyses, we have also purified the suitable 218 STR loci with four basepair repeat units and 53 loci with five basepair repeat units both for short read sequencing and PCR based technologies, which would be candidates to the actual forensic DNA typing in Japanese population.

## 1 Introduction

Forensic DNA typing is widely used to confirm the identification of missing persons in large-scale disasters and also plays a central role in legal profiling [1]. It uses PCR amplification, followed by capillary electrophoresis (CE) fragment analysis to detect the length of short tandem repeat (STR) markers [2, 3]. The AmpFlSTR™ Identifiler™ PCR Amplification Kit (Thermo Fisher Scientific, San Francisco, CA), which is mainly used for forensic DNA typing, examines fifteen STR loci [4]. For forensic DNA typing, allele frequencies should be diverse in the target population. Some loci have a low power of discrimination (PD) in the population. For example, the major allele frequency of TPOX is 0.45 in the Japanese population [5, 6], and this locus is also known to have a low PD in the Caucasian population [7] as well as in the Chinese population [8]. In most cases of forensic DNA typing, the accuracy of personal identification with the above kit is sufficient. However, it is difficult to distinguish close relatives due to genetic similarities [9, 10]. In recent years, DNA typing kits with a larger number of loci, such as GlobalFiler® PCR Amplification Kit (Thermo Fisher Scientific) [11] and PowerPlex® Fusion System (Promega, Madison, WI) [12], have become available, leading to more accurate sibship tests [13]. The reason for this is that accuracy generally improves as the number of examined STR loci increases [14, 15].

Thus, finding highly polymorphic loci for regional population typing is necessary [16]. More polymorphic STRs in regional populations, such as the D6S1043 locus in Asia [17, 18], allow higher precision in DNA typing.

In recent years, massively parallel sequencing (MPS) technologies have greatly advanced. MPS technologies are beginning to be applied to STR analyses, even in forensic science [19, 20, 21, 22]. STR analysis with MPS firstly involves detecting the flanking regions after aligning the sequenced reads to the reference assembly. Then it estimates the repeat counts with the flanking reads around the repetitive regions [23, 24].

Unlike traditional methods like capillary electrophoresis, MPS-based analysis directly identifies the nucleotide bases of the repeat units, and therefore measures not only the STR repeat number but also the variants in the STR region [25, 26].

In this study, we investigate highly polymorphic STR loci in Japanese individuals using an STR analysis tool to examine the MPS data obtained from 1,070 Japanese individuals (1KJPN) recruited in a prospective cohort study [27] and catalog the STR profile of the Japanese population (1KJPN-STRs).

While highly accurate DNA typing is possible by looking at the difference in nucleotide sequence within the STR sequence by MPS data, in this study we focused only on the length of STR and screened for highly polymorphic STR loci to enable DNA typing with both MPS and CE-based technology.

To evaluate the performance of 1KJPN-STRs, we compared the allele frequencies of 1KJPN-STRs to those of the STR test kit in Japanese individuals. The results reveal both advantages and disadvantages of an MPS-based approach.

Using the evaluated results and polymorphic scores, e.g. heterozygosity (HZ), polymorphic information content (PIC) [28], and Power of discrimination (PD) [29], we identify STR loci that could be applied to STR typing.

## 2 Materials and methods

### 2.1 Materials

We evaluated the performance of an available STR analysis tool to estimate the repeat units at STR loci in whole genome sequencing data. Japanese whole genome sequencing results were compared to data obtained with commercially available kits using CE-based technology as the reference.

#### 2.1.1 1KJPN

In this paper, we used high-coverage whole-genome sequence data from 1,070 Japanese individuals generated by massively parallel short-read sequencing (hereafter referred to as 1KJPN); Nagasaki et al. conducted the whole-genome sequencing of these individuals and published the original analysis of that data set [30]. These individuals recruited as part of a prospective cohort study at the Tohoku University Tohoku Medical Megabank Organization (ToMMo) with the approval of the ethics committee of the Tohoku University School of Medicine [27].

Data were obtained by sequencing using the standard PCR-free protocol by reading DNA fragments with a mean length of 550 bp, inserted between 162 bp paired-end reads [30, 31].

#### 2.1.2 1.5K-NRIPS

Allele frequencies of 1,501 Japanese individuals previously obtained at the National Research Institute of Police Science (NRIPS) using GlobalFiler® PCR Amplification Kit [6] (Thermo Fisher Scientific) and PowerPlex® Fusion System [5] (Promega) kits were used as the reference allele frequencies for the Japanese population (hereafter referred to as 1.5K-NRIPS) for the following 23 commonly used STR loci (hereafter referred to as CU23STRs): D1S1656, TPOX, D2S441, D2S1338, D3S1358, FGA, D5S818, CSF1PO, SE33, D7S820, D8S1179, D10S1248, TH01, vWA, D12S391, D13S317, PentaE, D16S539, D18S51, D19S433, D21S11, PentaD, and D22S1045. However, the DNA samples of these individuals were not available for analysis in this study.

### 2.2 Methods

#### 2.2.1 Analysis of 23 commonly used STR loci in 1KJPN

For 1KJPN, index files including a custom reference set of the CU23STRs were prepared for analysis by the STR analysis software lobSTR, version 3.0.3 [23]. The CU23STRs of 1KJPN were analyzed using the default parameters of lobSTR to obtain the allele frequencies of each STR locus.

lobSTR was selected among STR detection tools for two reasons. Firstly, the tool is well-maintained and has been developed and evaluated by many researchers [32, 33, 34]. Secondly, the data used for this study (1KJPN) is high-coverage sequencing data (32.4x), and lobSTR has demonstrated high performance for high-coverage sequencing data (more than 30x) compared with popSTR, another STR analysis tool [35]. For the 1KJPN STR analysis (hereafter referred to as 1KJPN-23STRs), allele and genotype frequencies, observed heterozygosity (obs-HZ), expected heterozygosity (exp-HZ), and Hardy-Weinberg Equilibrium probability (HWE-p) exact test were calculated using Genepop version 4.5.1 [36]. Additionally, we calculated the PD [29] from the obtained genotype frequencies (P_i_) by using the following formula.

$$\text{PD}=1-\sum_{\text{i}=1}^{\text{m}}{{\text{P}}_{\text{i}}}^{2}$$

#### 2.2.2 Comparison between 1KJPN-23STRs and 1.5K-NRIPS

The DNA materials of the individuals in 1KJPN and 1.5K-NRIPS were not available in this study and thus we could not directly compare the repeat numbers for the same Japanese individual. However, given that the proportion of non-Japanese in Japan is very small, allele frequencies are more predictable. This allowed us to compare the available frequency distribution of STR repeat numbers in 1.5K-NRIPS [5, 6] and the estimated result from MPS in 1KJPN.

We calculated the correlation coefficients of the allele frequencies between 1KJPN-23STRs and 1.5K-NRIPS. Before the comparison, at D2S1338, D19S433, and D21S11 loci, the repeat numbers of 1KJPN-23STRs were calibrated based on the repeat number of the human genome reference build hg19. The details of the calibrations and their analyses are described in Section 3.3.

#### 2.2.3 Construction of STR catalog in 1KJPN

For the 1KJPN reference panel, the 843,473 candidate STR loci with two to six basepair repeats provided from the official lobSTR website were analyzed with lobSTR v3.0.3., using the same protocol as that used for the CU23STRs.

We extracted all three, four, and five basepair repeat units from the VCF result from lobSTR. For the selected STR loci, we calculated allele and genotype frequencies, obs-HZ, exp-HZ, and HWE-p using GenePop version 4.5.1., as well as the PD from obtained genotype frequencies. From these STR loci, we selected loci with 0.8 < obs-HZ, 0.8 < exp-HZ and call rate = 1. Here, call rate is defined as follows: the total number of samples with STR repeat units identified with lobSTR, divided by the total number of samples. We followed the nomenclature of STR loci of the International Society for Forensic Genetics [37].

## 3 Results and discussion

### 3.1 The call rate of 1KJPN-23STRs

Fifteen loci had very high call rates (>0.99). These loci were CSF1PO, D10S1248, D13S317, D22S1045, D2S441, D5S818, D7S820, D8S1179, FGA, TH01, TPOX, D3S1358, D18S51, D19S433, and PentaD (ordered by call rate; Table 1). Two loci, D2S1338 and vWA, had high call rates (>0.95). The other six loci, PentaE: 0.873, D16S539: 0.759, SE33: 0.733, D21S11: 0.285, D1S1656: 0.140, and D12S391: 0.088, had low call rates (ordered by call rate; Table 1).

**Table 1:** The call rates of 1KJPN-23STRs and correlation coefficients between 1KJPNSTR and 1.5K-NRIPS. The STRs ordered according to their call rates. (Call rate: for each STR loci, the total number of samples with STR repeat units identified with lobSTR, divided by the total number of samples.)

	Call rate	Correlation coefficient
CSF1PO	1.000	0.9991
D10S1248	1.000	0.9922
D13S317	1.000	0.9989
D22S1045	1.000	0.9968
D2S441	1.000	0.9978
D5S818	1.000	0.9979
D7S820	1.000	0.9991
D8S1179	1.000	0.9922
FGA	1.000	0.9663
TH01	1.000	0.9959
TPOX	1.000	0.9968
D3S1358	0.999	0.9981
D18S51	0.998	0.9959
D19S433	0.998	0.9993
PentaD	0.994	0.9954
D2S1338	0.985	0.9841
vWA	0.969	0.8904
PentaE	0.873	0.8079
D16S539	0.759	0.9983
SE33	0.733	0.7088
D21S11	0.285	0.6956
D1S1656	0.140	0.3762
D12S391	0.088	0.3361

### 3.2 Comparison of 1KJPN-23STRs and 1.5K-NRIPS

**Fig. 1:**
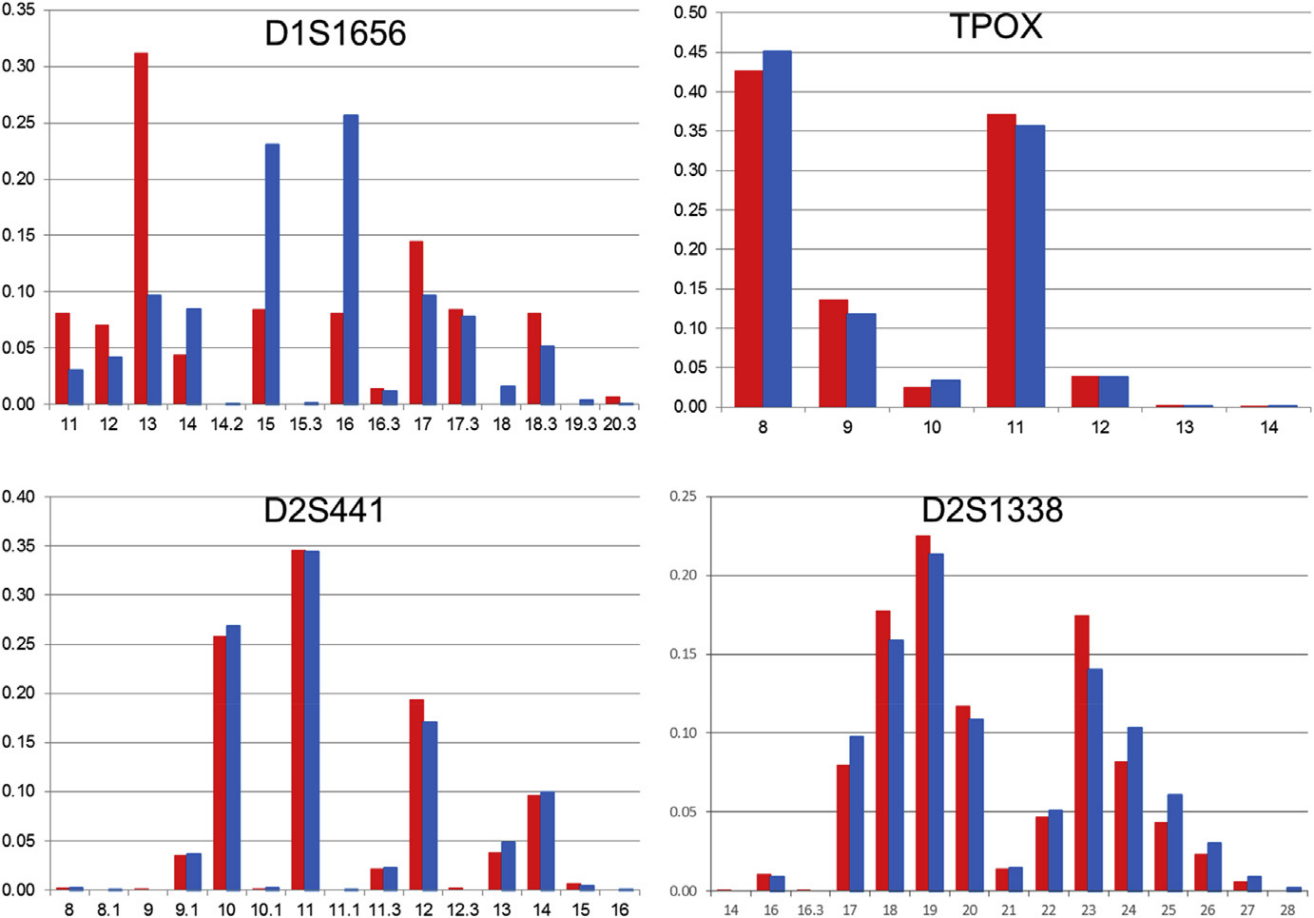
The comparison of allele frequencies of STR loci on chromosome 1 and 2 in 1KJPN-23STRs and 1.5K-NRIPS. Red bars represent 1KJPN-23STRs, and blue bars represent 1.5K-NRIPS. Horizontal axis: repeat unit. Vertical axis: frequency.

**Fig. 2:**
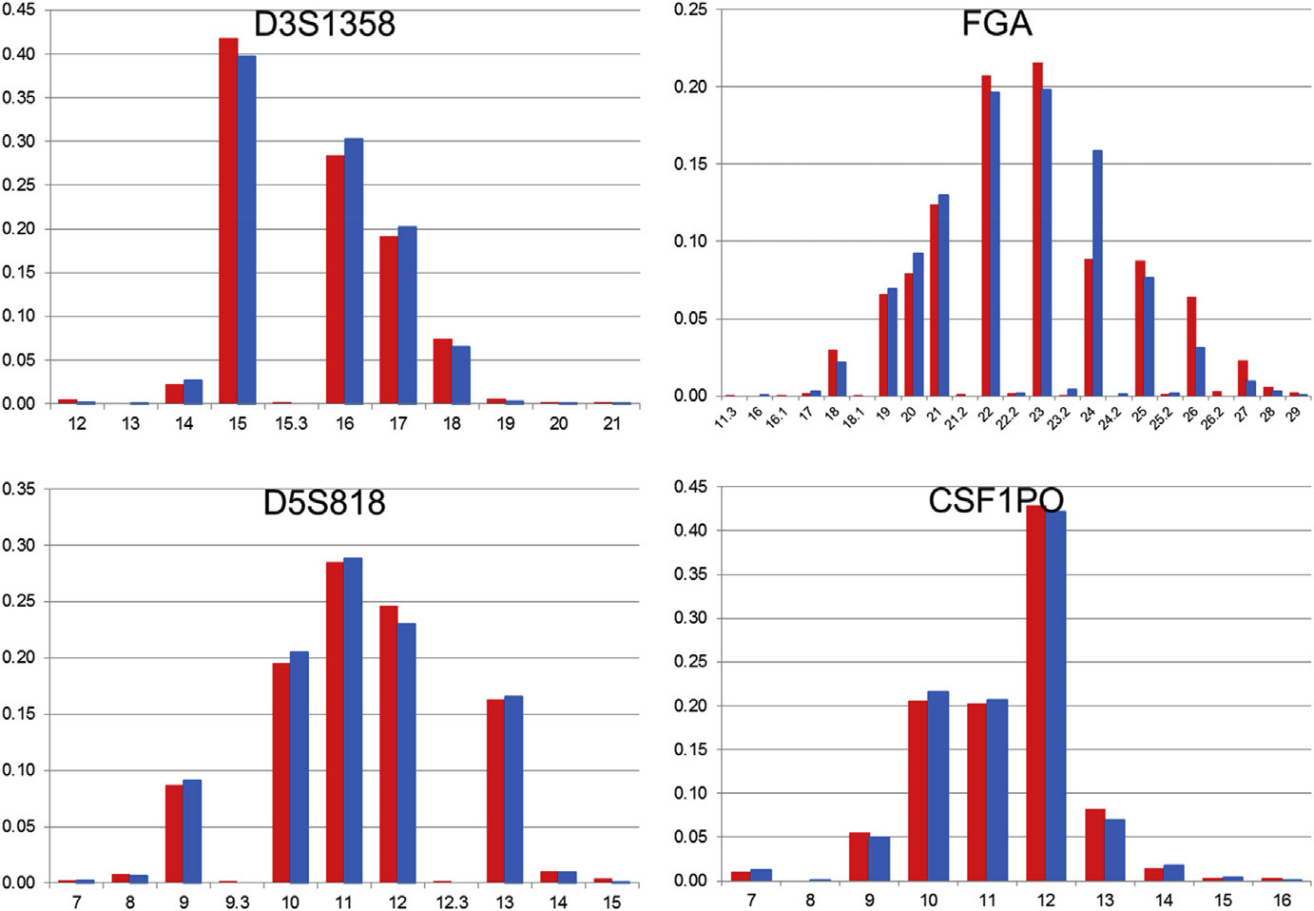
The comparison of allele frequencies of STR loci on chromosome 3, 4, and 5 in 1KJPN-23STRs and 1.5K-NRIPS. Red bars represent 1KJPN-23STRs, and blue bars represent 1.5K-NRIPS. Horizontal axis: repeat unit. Vertical axis: frequency.

**Fig. 3:**
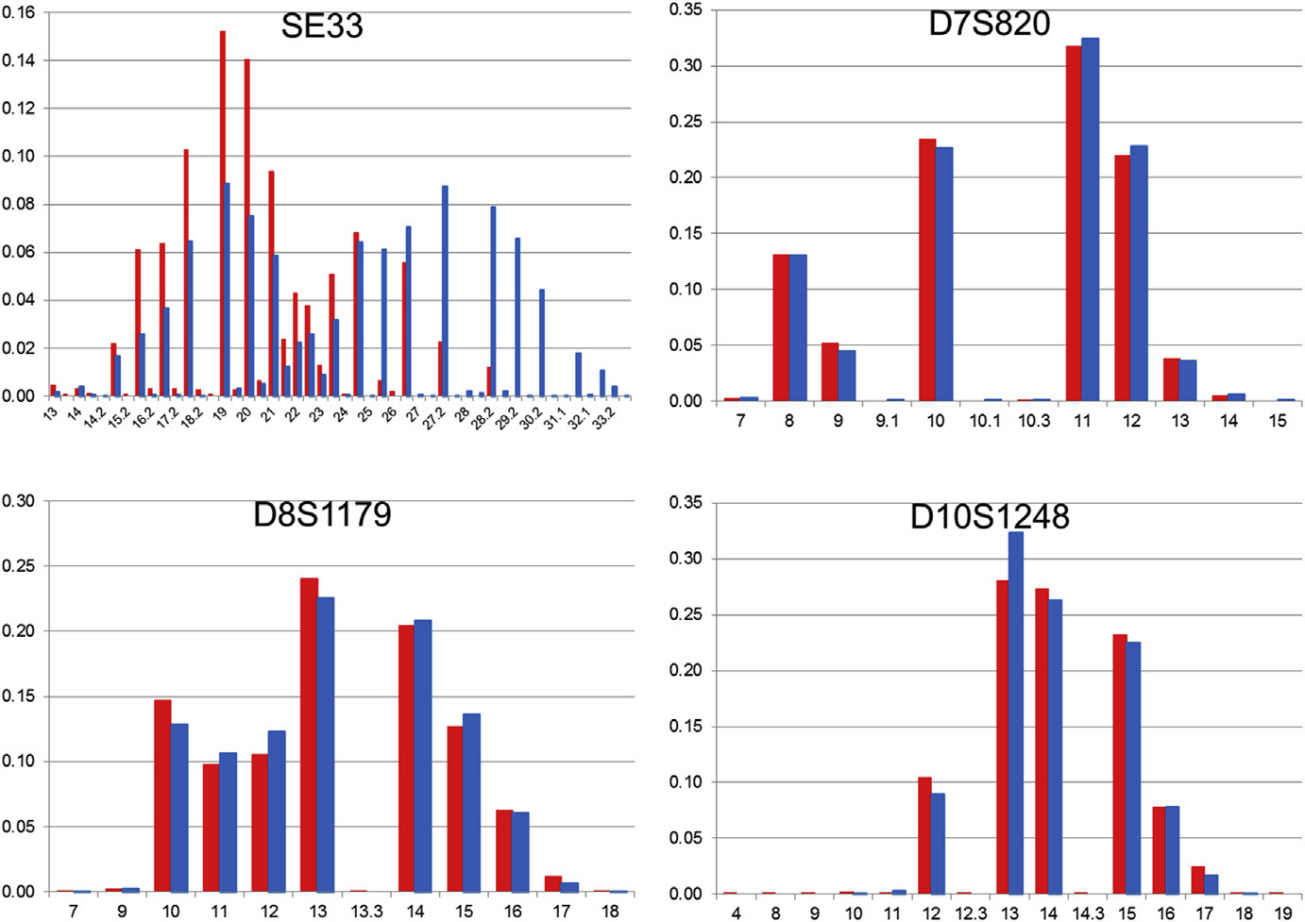
The comparison of allele frequencies of STR loci on chromosome 6, 7, 8, and 10 in 1KJPN-23STRs and 1.5K-NRIPS. Red bars represent 1KJPN-23STRs, and blue bars represent 1.5K-NRIPS. Horizontal axis: repeat unit. Vertical axis: frequency.

**Fig. 4:**
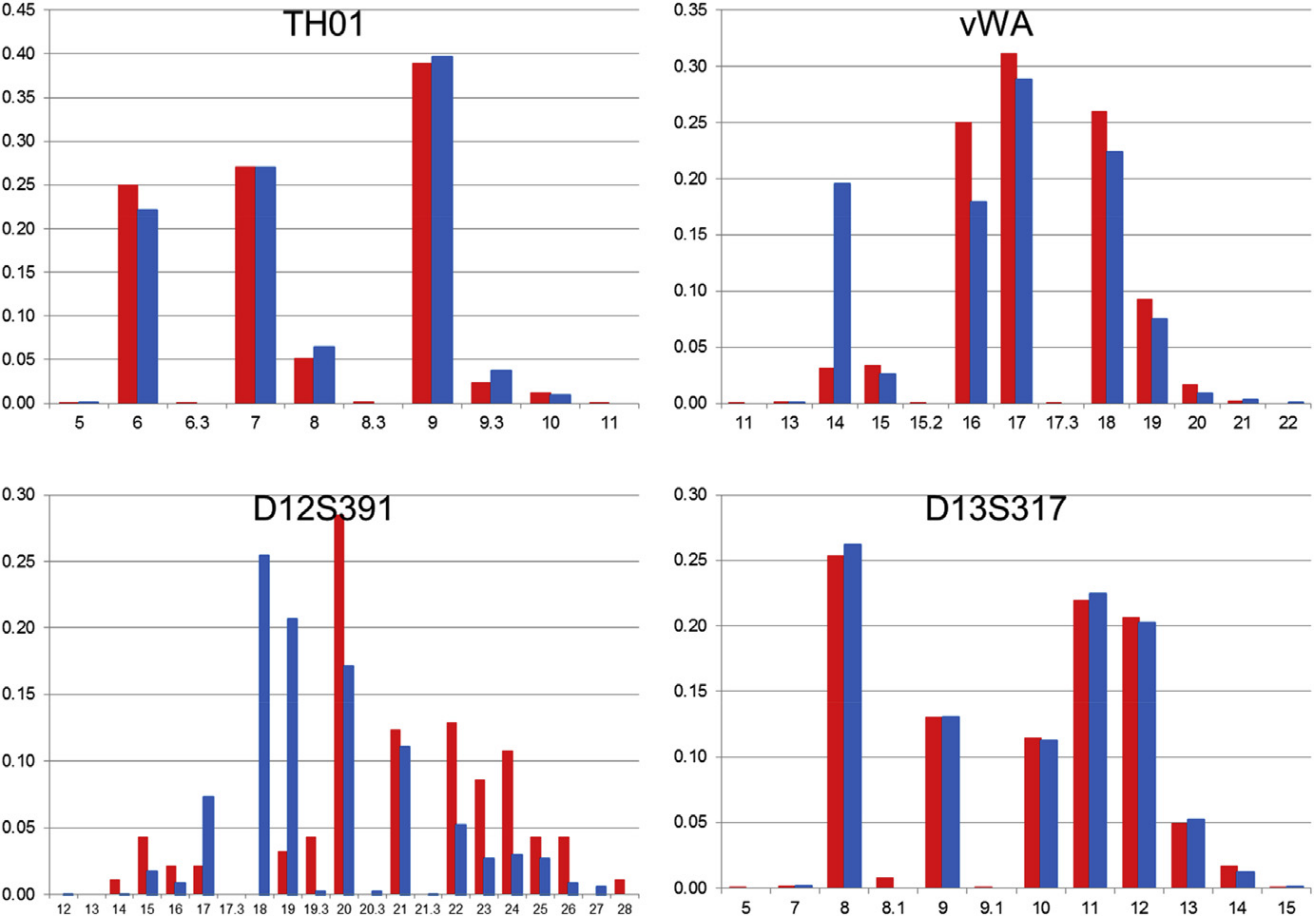
The comparison of allele frequencies of STR loci on chromosome 11, 12, and 13 in 1KJPN-23STRs and 1.5K-NRIPS. Red bars represent 1KJPN-23STRs, and blue bars represent 1.5K-NRIPS. Horizontal axis: repeat unit. Vertical axis: frequency.

**Fig. 5:**
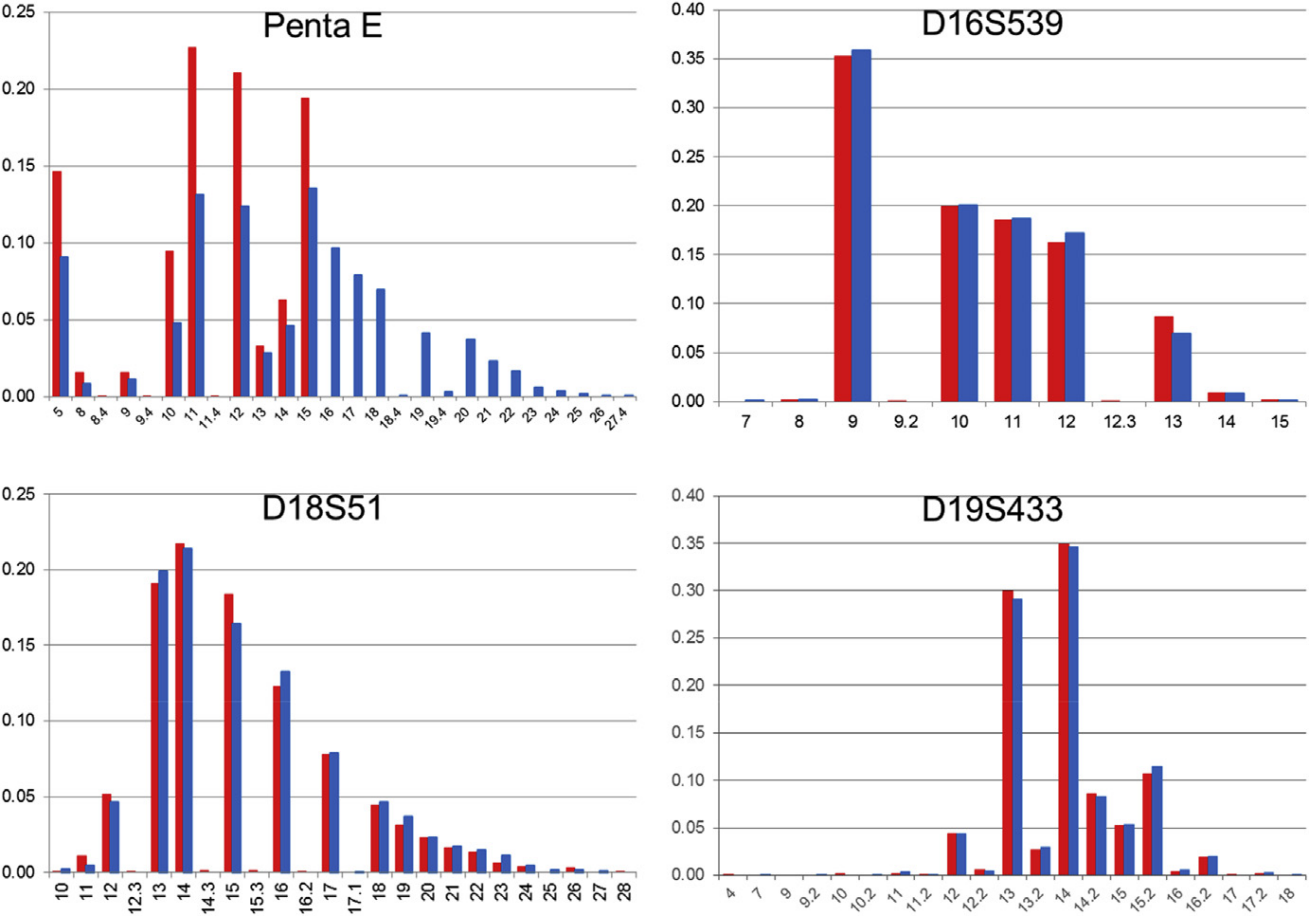
The comparison of allele frequencies of STR loci on chromosome 15, 16, 18, and 19 in 1KJPN-23STRs and 1.5K-NRIPS. Red bars represent 1KJPN-23STRs, and blue bars represent 1.5K-NRIPS. Horizontal axis: repeat unit. Vertical axis: frequency.

**Fig. 6:**
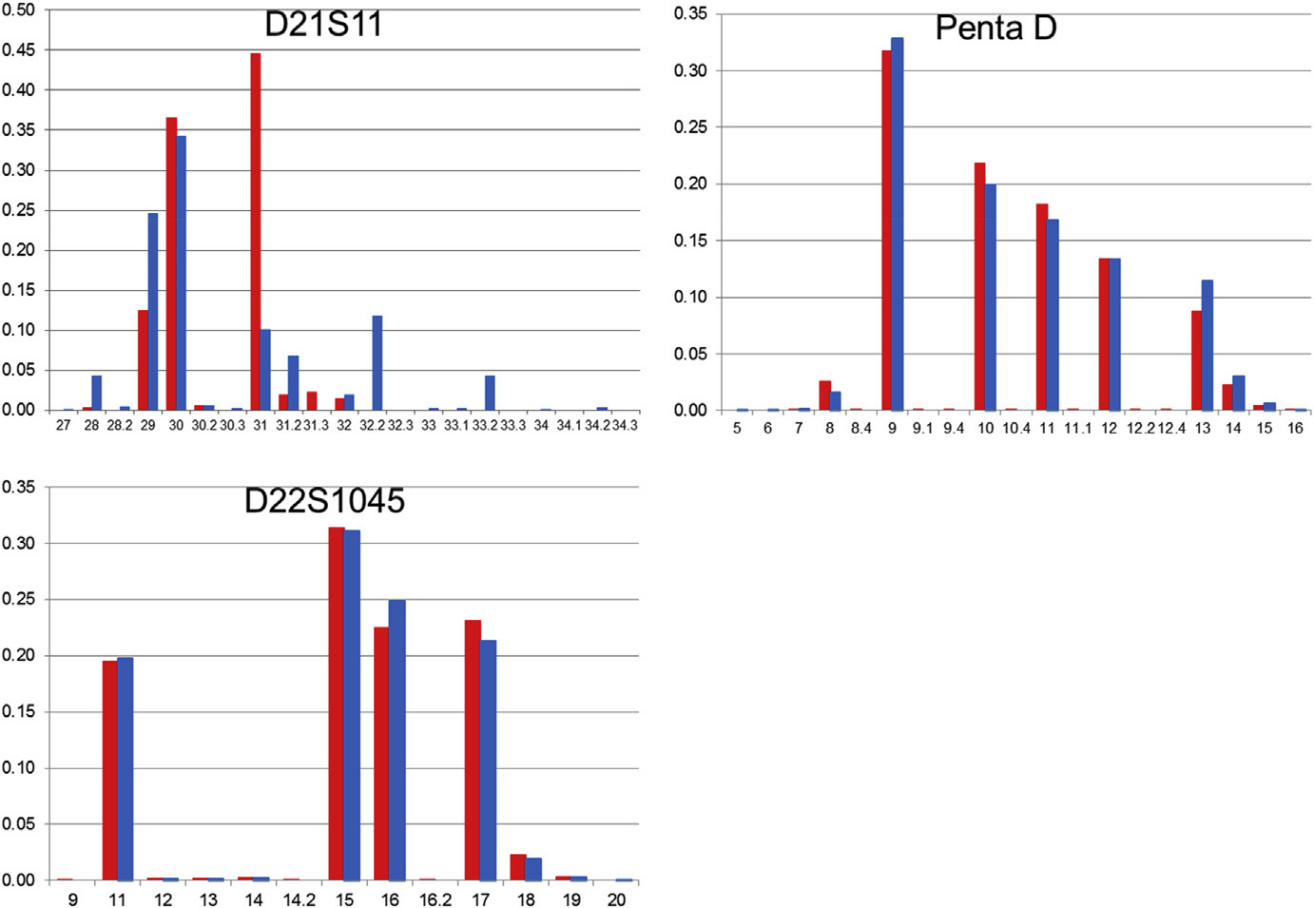
The comparison of allele frequencies of STR loci on chromosome 21 and 22 in 1KJPN-23STRs and 1.5K-NRIPS. Red bars represent 1KJPN-23STRs, and blue bars represent 1.5K-NRIPS. Horizontal axis: repeat unit. Vertical axis: frequency.

1KJPN-23STRs and 1.5K-NRIPS results are compared in Figs. 1, 2, 3, 4, 5, and 6. All loci with very high call rates (>0.99) in 1KJPN-23STRs, except for FGA (0.9663), showed high correlations in allele frequencies between 1KJPN-23STRs and 1.5K-NRIPS (correlation coefficient > 0.99). Furthermore, in these loci, the difference between exp-HZ and obs-HZ was small (Supplementary Table 1).

Table 1 and Supplementary Fig. 1 show the relationship between the call rate and correlation coefficient of 1KJPN-23STRs and 1.5K-NRIPS. Supplementary Fig. 1 clearly displays the positive correlation between call rate and allele frequency correlation between 1KJPN-23STRs and 1.5K-NRIPS.

Seven loci had call rate under 0.98. The call rate of the D16S539 locus was not high (0.7585), in contrast the correlation to the called samples was very high (0.9983; Table 1 and Supplementary Fig. 1). In some cases, lobSTR is unable to determine repeat numbers for some samples; since these are not called, this causes the call rate to decrease. However, because the remaining samples that are called accurately, this decline in call rate is not necessarily associated with a decrease in correlation.

The other six loci, vWA: 0.8904, PentaE: 0.8079, SE33: 0.7088, D21S11: 0.6956, D1S1656: 0.3762, and D12S391: 0.3361, showed low allele frequency correlations (Table 1 and Supplementary Fig. 1).

### 3.3 Inconsistent loci between 1KJPN-23STRs and 1.5K-NRIPS

Two loci, SE33 and PentaE, showed especially low correlations between 1KJPN-23STRs and 1.5K-NRIPS in regions of many repeats, e.g. n ≥ 24 in SE33 and n ≥ 16 in PentaE (Fig. 5). This is thought to be owing to the length of the repeats exceeding the length of one read with the MPS technology, i.e. 162 bases. Thus, the tool failed to calculate the true repeat counts for these individuals, resulting in a no call.

In PCR-based technology, the target region must be amplified from the primer region and cover the whole region of the STR loci. Usually, as the primer region is located relatively far away from the STR loci, the total length of the amplified region tends to be somewhat longer than the total length of STR repeat numbers. Instead, MPS just requires the minimum unique sequence bases, e.g. from 15 to 30 bases, for both ends of STR loci. In our study, each sequence read is 162 bases long, and a maximum of approximately 34, 25, and 20 repeat numbers can be calculated in the case of three, four, and five repeat unit case, respectively.

Three other loci, i.e. D1S1656, FGA, and vWA, with low correlations between 1KJPN-23STRs and 1.5K-NIPS data had complicated repeat patterns, [TAGA]_a_ [TGA]_(0,1)_ [TAGA]_b_ [TAGG]_(0,1)_ [TG]_5_, [GGAA]_a_ [GGAG]_1_ [AAAG]_b_ [AGAA] [AAAA] [GAAA]_c_ and [TCTA]_a_ [TCTG]_b_ [TCTA]_c_, respectively. This complexity seemed to divide the estimated repeat counts from lobSTR and caused inconsistencies between the estimated results in 1KJPN-23STRs and those in 1.5K-NRIPS.

Two loci, D2S1338 and D19S433, had high correlations, but the repeat counts were different when comparing the estimated results in 1.5K-NRIPS and 1KJPN-23STRs.

At the D2S1338 locus, 1.5K-NRIPS calculated the [TGCC]_a_ [TTCC]_b_ repeats from the reverse strand [25] (23 times in the human genome reference build hg19, Supplementary Fig. 2). On the other hand, in the 1KJPN-STRs, the number of repeats of [AGGA] in the forward strand were counted (17.3 times in the human genome reference build hg19, Supplementary Fig. 2). Thus, a difference of 5.1 repeats occurred (21 bases equal to five repeats of four STR unit and one base). At the D19S433 locus, similar to the D2S1338 locus, a difference of one repeat occurred (Supplementary Fig. 3) [37]. By taking into account the difference between the forward and reverse strands for the D2S1338 and D19S433 loci, the number of repeats in 1KJPN-23STRs was correctly calibrated to 1.5K-NRIPS (D2S1338 plots in Fig. 1 and D19S433 plots in Fig. 5).

Supplementary Fig. 4 shows the D21S11 locus with a complex repeating structure, [TCTA]_a_ [TCTG]_b_ [TCTA]_c_ TA [TCTA]_d_ TCA [TCTA]_e_ TCCATA [TCTA]_f_ [38]. In 1.5K-NRIPS, the four bases enclosed by [TCTG] and [TCTA] were counted as the number of repeats, i.e. a + b + c + d + e + f. The eleven underlined bases were not included. However, 1KJPN-STRs also included these eleven bases, thus there was a difference in the number of repeats between 1KJPN-STRs and 1.5K-NRIPS by 2.3. However, taking into account this difference, the difference of frequencies between 1KJPN-STRs and 1.5K-NRIPS still remained (D21S11 plots in Fig. 6). The result would be due to the complex repeat pattern of the D21S11 locus. The estimate could be also confirmed by the low call rate of D21S11 in 1KJPN-23STRs (Table 1). Thus, we concluded that the D21S11 locus was difficult to analyze using lobSTR.

The overview of the lobSTR algorithm is as follows. First, the tool tries to detect STRs from informative sequencing reads and determines the repeat pattern. Secondly, the tool aligns the STRs' flanking regions to the reference genome. This step limits the detectable STR repeat counts to the length of each sequenced read, i.e. 162 bases in 1KJPN. SE33 and PentaE were matched in our case. Thirdly, the tool infers the allelotype and variations in the allelotype. In the third step, although to some degree lobSTR takes into account small variations in a given repeat unit sequence, it would be difficult to infer the true allelotype in STRs with complex repeats, e.g. D21S11, which has many repeat patterns. These three steps are repeated for all potential STR loci.

### 3.4 Novel highly polymorphic STRs in 1KJPN-STRs

The above comparison between 1KJPN-23STRs and 1.5K-NRIPS suggested that the 1KJPN-STRs would have many STR loci with better performance than CU23STRs in the Japanese population.

**Fig. 7:**
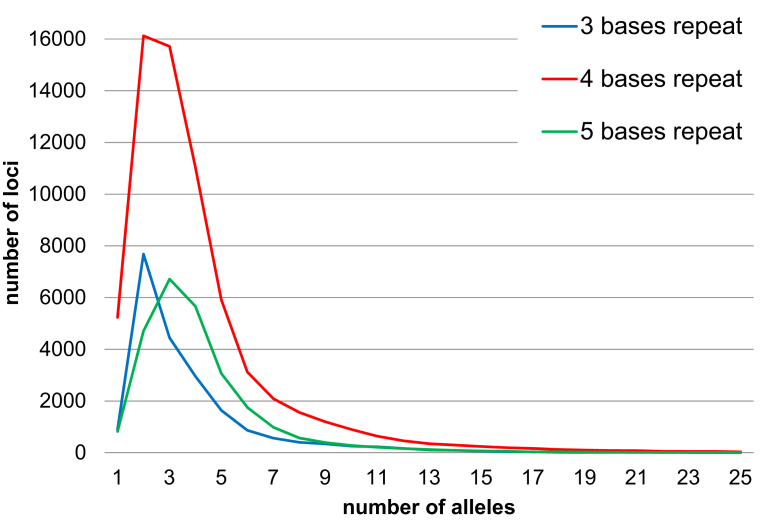
The number of variations of each STR loci in 1KJPN-STRs. Horizontal-axis: The number of alleles to the target STR loci. Vertical-axis: The number of loci in 1KJPN-STRs with the call rate >0.

Fig. 7 summarizes the number of STR loci with three to five basepair repeat units that have allelic variations in the 1,070 Japanese individuals (STRs with three basepair repeat units, 84,869; four basepair repeat units, 262,179; and five basepair repeat units, 106,418). For the following reasons, we have excluded STR loci with two and more than six basepair repeat units.

Firstly, to be adopted for PCR based STR typing, it is best to avoid STR loci with two basepair repeat units, since they are susceptible to a PCR by-product known as stutter product [39, 40]. This occurs at the PCR amplification step and involves slipping backwards one repeat unit. With longer repeat units, stutter product frequency tends to decrease.

Secondly, the DNA in forensic samples is often degraded and fragmented [41] and therefore shorter STR regions with high variability are desired [42, 43]. Thus, it is difficult to use loci with more than six base repeats for STR typing, since the PCR amplification products tend to be long.

Table 2 shows that the STR loci with four basepair repeat units had the maximum number of loci (60,780) compared to the STR loci with three (19,960) or five (25,004) basepair repeat units with the maximum call rate one.

**Table 2:** Summary of the total number of STR loci detected by using Tandem repeats finder and the total number of loci with call rate = 1 in 1KJPN-STRs.

	Number of detected STR loci by using Tandem repeats finder	The number of STR loci with call rate = 1	Proportion (%)
3 bases repeats	84,869	19,960	23.50
4 bases repeats	262,179	60,784	23.20
5 bases repeats	106,418	25,004	23.50

Table 3 shows that the number of passed STR loci in each filtering steps. To choose the STR loci that were highly polymorphic in this Japanese population, we used the following five criteria; i) in autosomal regions, ii) four or five basepair repeats, iii) five or more alleles, iv) expected heterozygosity and observed heterozygosity both exceeding 0.8, and v) call rate equal to 1. These conditions were determined based on the high performance STRs in CU23STRs. As a result, we identified novel 218 autosomal STR loci with four basepair repeat units and 53 loci with five basepair repeat units that were highly polymorphic and had high call rates in the Japanese population. As a precondition, the 271 candidate (novel) STR loci were estimated from MPS with 162 bp in length. Since long repetitive sequences (STRs) that exceed 162 bp do not meet criteria (iv) or (v) above and would therefore be filtered out, we did not apply the explicit STR repeat length filtering in our filtering steps.

**Table 3:** The number of passed STR loci in each filtering step.

	Step2 analysed with call rate > 0	Step3 more than 5 alleles	Step4 obsHZ > 0.8 and expHZ > 0.8	Step5 call rate = 1
chr1	30497	3207	45	28
chr2	28481	2954	44	26
chr3	23829	2518	37	21
chr4	21853	2299	41	26
chr5	20378	2153	28	16
chr6	20648	2256	36	22
chr7	20100	2225	26	14
chr8	18016	1923	27	15
chr9	14366	1546	29	16
chr10	17170	1870	16	5
chr11	16332	1659	14	8
chr12	18285	1965	31	16
chr13	11250	1231	20	9
chr14	11293	1193	25	14
chr15	10224	1041	6	6
chr16	12689	1302	12	3
chr17	13057	1409	15	6
chr18	9054	1011	10	6
chr19	12731	1390	3	0
chr20	9004	942	14	9
chr21	4358	567	9	5
chr22	5757	638	4	0
Total	349372	37299	492	271

The 271 STR loci and their statistics are shown in the Supplementary Tables (2–23), and the global map of these 218 STR loci with four basepair repeat units in Supplementary Fig. 5.

## 4 Conclusions

We analyzed 843,473 STR candidate loci in Japanese individuals and cataloged polymorphic STRs in the Japanese population using high-coverage human whole genome data of 1,070 individuals. The allele frequencies of CU23STRs were evaluated by comparing to those obtained using a commercial kit with PCR-based technology in Japanese individuals. Results at many of the STR loci were consistent between data sets, except for six STR loci with highly complex patterns or with many STR repeats. These results indicated that many STR loci could be typed using whole-genome short read sequencing technology.

We also focused on the STR loci with four and five basepair repeat units and selected 218 and 53 loci highly polymorphic in Japanese with five conditions suitable for both standard PCR-based and new MPS based technologies, which would be applicable to actual STR typing.

The current limitation of our protocol of short-read sequencing is that less than 162 bases are sequenced in one read. The maximum length of the repeat units in the 271 STR loci is always shorter than that length and so this technique is valid. With the future advancement of sequencing technology, the limitation of length might be relaxed. However, many forensic samples contain fragmented DNA and this factor should be always considered.

In future work, we will investigate the feasibility of conducting PCR amplification of the 218 four basepair repeat units and 53 five basepair repeat units STR loci selected in this study using immortalized lymphocytes from Japanese individuals. The validated loci will then be further evaluated for use in actual STR typing.

## Declarations

### Author contribution statement

Satoshi Hirata, Masao Nagasaki: Conceived and designed the experiments; Performed the experiments; Analyzed and interpreted the data; Wrote the paper.

Kaname Kojima: Conceived and designed the experiments; Performed the experiments; Analyzed and interpreted the data.

Kazuharu Misawa, Olivier Gervais: Analyzed and interpreted the data; Wrote the paper.

Yosuke Kawai: Conceived and designed the experiments; Analyzed and interpreted the data.

### Funding statement

This work was partially supported by grants from the Reconstruction Agency, the Ministry of Education, Culture, Sports, Science and Technology (MEXT), the Japan Agency for Medical Research and Development (AMED) (JP17km0405001), the Platform Program for Promotion of Genome Medicine (JP17km0405205), and the Center of Innovation Program from Japan Science and Technology Agency (JST).

### Competing interest statement

The authors declare no conflict of interest.

### Additional information

No additional information is available for this paper.
