# Supplementary material for: Population-scale whole genome sequencing identifies 271 highly polymorphic short tandem repeats from Japanese population
Source: Heliyon. 2018 May 22;4(5):e00625. doi: 10.1016/j.heliyon.2018.e00625 (PMC5986539; doi:10.1016/j.heliyon.2018.e00625)
Supplement: Supplementary Fig 1 [file mmc1.docx]

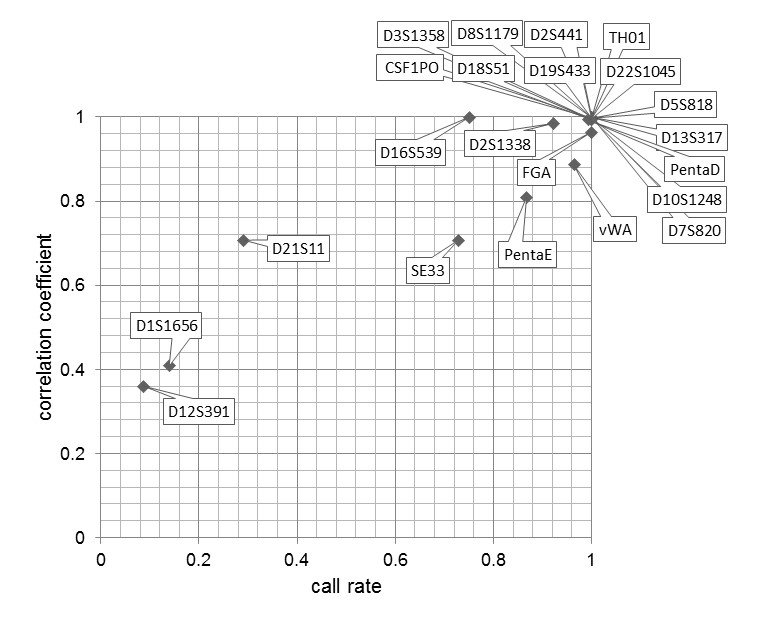
 **Supplementary Fig. 1** The relationship between the call rate of 1KJPN-23STRs (horizontal-axis) and the correlation coefficient of the allele frequencies of 1KJPN-23STRs and 1.5K-NRIPS (vertical-axis).
