# Supplementary material for: Population-scale whole genome sequencing identifies 271 highly polymorphic short tandem repeats from Japanese population
Source: Heliyon. 2018 May 22;4(5):e00625. doi: 10.1016/j.heliyon.2018.e00625 (PMC5986539; doi:10.1016/j.heliyon.2018.e00625)
Supplement: Supplementary Fig 2 [file mmc2.docx]

**
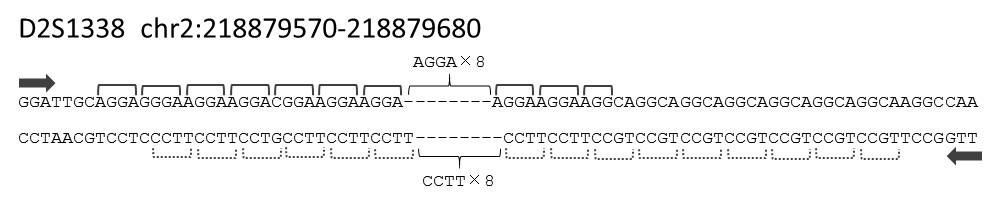
**

**Supplementary Fig. 2** A repeat structure of the D2S1338 locus. The number of repeats was determined by counting the AGGA repeat unit in the forward chain by using the STR estimate software (17.3 repeats). In contrast, the number [TGCC]_a_ [TTCC]_b_ repeats were determined in the reverse chain by using a commercially available kit (23 repeats), where a and b were variables that differed among individuals.
