# Supplementary material for: Population-scale whole genome sequencing identifies 271 highly polymorphic short tandem repeats from Japanese population
Source: Heliyon. 2018 May 22;4(5):e00625. doi: 10.1016/j.heliyon.2018.e00625 (PMC5986539; doi:10.1016/j.heliyon.2018.e00625)
Supplement: Supplementary Fig 3 [file mmc3.docx]

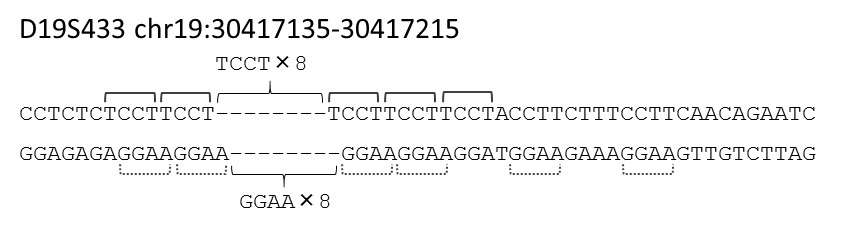


**Supplementary Fig. 3** A repeat structure of the D19S433 locus. The number of TCCT repeat units were determined in the forward chain by using the STR estimate software (13 repeats). In contrast, the number of AAGG repeat units were determined in the reverse chain by using a commercially available kit (14 repeats).
