# Supplementary material for: Population-scale whole genome sequencing identifies 271 highly polymorphic short tandem repeats from Japanese population
Source: Heliyon. 2018 May 22;4(5):e00625. doi: 10.1016/j.heliyon.2018.e00625 (PMC5986539; doi:10.1016/j.heliyon.2018.e00625)
Supplement: Supplementary Fig 4 [file mmc4.docx]

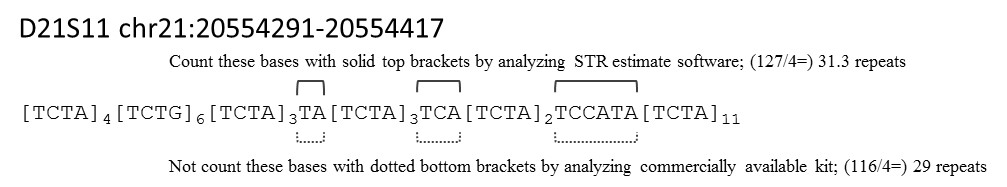


**Supplementary Fig. 4** A repeat structure of the D21S11 locus. The number of repeats, including non-repetitive bases, was determined by using the STR estimate software (31.3 repeats). In contrast, the number of repeats, without including non-repetitive bases, was determined by using a commercially available kit (29 repeats).
