# Supplementary figures and images for: Population-scale whole genome sequencing identifies 271 highly polymorphic short tandem repeats from Japanese population
Source: Heliyon. 2018 May 22;4(5):e00625. doi: 10.1016/j.heliyon.2018.e00625 (PMC5986539; doi:10.1016/j.heliyon.2018.e00625)

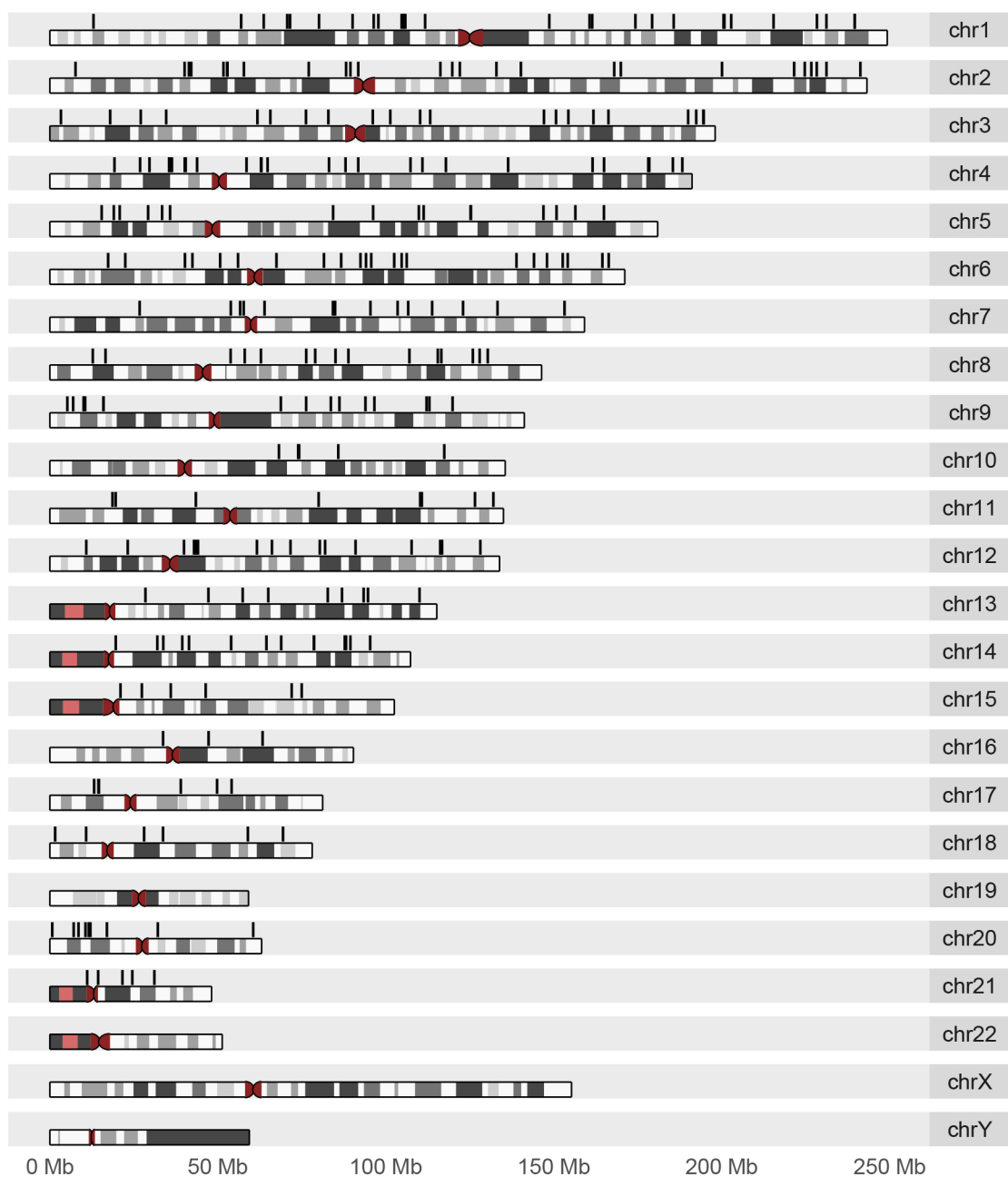

Supplementary Fig. 5

The chromosome location of the selected 271 STR loci in Japanese population.

Supplement: Supplementary Fig 5 [file mmc5.pdf]
